# Preparation and evaluation of some nanocarbon (NC) based composites for optoelectronic applications

**DOI:** 10.1038/s41598-023-35754-9

**Published:** 2023-06-02

**Authors:** Noha Elhalawany, Amal M. Abdel-karim, Maher M. Saleeb

**Affiliations:** 1 Polymers and Pigments Department, National Research Centre, 33 El Bohouth St. Dokki, Giza, 12622 Egypt; 2 Physical Chemistry Department, National Research Centre, 33 El Bohouth St. Dokki, Giza, 12622 Egypt; 3 Eagle Chemicals Company, 6th of October City, Egypt

**Keywords:** Chemistry, Materials science

## Abstract

Polyaniline/nanocarbon (PANI/NC) nanocomposites have been prepared by in situ polymerization of aniline monomer in the presence of a stable colloidal solution of nanocarbon NC using ammonium persulfate as an initiator and silver ions (Ag^+^) as oxidizing agents to produce PANI/NC and PANI/NC/Ag_2_O nanocomposites, respectively. The morphological studies of the formed nanocomposites have been elucidated via transmission and scanning electron microscopes (TEM and SEM). Further characterization of the prepared nanocomposites has been done via infrared spectroscopy (IR), X-ray diffractometer (XRD), X-ray photoelectron spectroscopy (XPS), particle size distribution analysis (PSD), fluorescence microscope (FM), UV–VIS spectroscopy, and finally surface analysis. XRD results confirmed the presence of silver oxide Ag_2_O nanoparticles, and the obtained data is well matched with the JCPDS card number 76–1393 of silver oxide. XPS analyses have shown two prevailing characteristic peaks for Ag 3*d*5*/*2 and Ag 3*d*3*/*2 at 367.1 and 373 eV, respectively, representing Ag_2_O nanoparticles, which are matchable with the XRD analysis. The PSD analysis revealed that the sizes of the prepared nanocomposites are in the size range from 60 to 140 nm. The FM measurements showed luminescence from the prepared nanocomposites upon irradiation with different lights. This recommends that the fluorophores present in the prepared nanocomposites have the potential to both absorb and emit light. The AC conductivity and the dielectric permittivity of the obtained nanocomposites at room temperature and at different frequency ranges have been investigated. At higher frequency ranges, the maximum ac conductivity obtained was 1.06 × 10^–2^ and 2.5 × 10^–2^ S.Cm^-1^ for the PANI/NC and PANI/NC/Ag_2_O, respectively. As far as we know, these new nanocomposites with superior optical and electrical characteristics have not been described yet in the literature.

## Introduction

The compounds known as polymeric nanocomposites contain nanoparticles like nanofillers. The nanofillers may be of many types and forms, but at least one of their sizes must fall below 100 nm. Soot, graphene, carbon nanotubes, and graphene may all be examples of nanofillers^1–3^.

The tight requirements of managing the filler's dispersion into the matrix and further stabilizing the resulting compound guide technologies for nanocomposite creation. Recently, research has been focused on carbonaceous nanofiller materials, like grapheme nanoplatelets (GNPs) and carbon nanotubes (CNTs)^4–6^. CNTs are a class of nanomaterials that provide a variety of uses because of their high aspect ratio, large surface area, and unique electrical, thermal, and optical properties^1,7^. CNTs are strong like a diamond while being light, thermally and electrically conductive, and formable like graphite.

One category of hybrid materials is called conducting polymer nanocomposites, which comprise of an inorganic element with at least one dimension in the nanoscale range and a polymer matrix (conductive polymer). Conducting polymer nanocomposites are a group of materials with significant applications in an assortment of electronic devices, such as light-emitting diodes, batteries, photovoltaics, and sensors^8–11^.

For the formation of the nanocomposites, a variety of preparation methods have been used, including the microemulsion, sol gel method, and chemical oxidative process^12^.

Conducting polymer nanocomposites have attracted the attention of numerous researchers due to their well-known unique optical and electrocatalytic properties. Molecules like dyes, carbazole compounds, and heterocyclic structures all have fast optical responsiveness, excellent chemical constancy, optical and nonlinear optical behaviors^13–15^. They have particularly substantial nonlinear optical properties as a result of the multiple -electron system conjugations in their structure.

Conducting polymers are currently widely used in the production of optical modulators and switches^16,17^. The outstanding optical and nonlinear optical (NLO) properties of polyaniline (PANI) have been established. This is due to its quick response, ease of production, and stability. ^18–20^.

PANI does, however, have disadvantages, such as poor mechanical characteristics and insolubility in typical organic solvents. The development of PANI characteristics can be accomplished through copolymerization and composite production^18,19,21–23^.

Considering its exceptional electrical, optical, and environmental durability, polyaniline (PANI) is the most impressive conductive polymer^24,25^. It has been hypothesized that adding metal^26^ or semiconductor nanoparticles^9,27,28^, CNT^4^ and graphene^5^ to any polymer matrix could improve its electrical and catalytic properties and its sensing capabilities.

In 2009, a team at Taiwan's Institute of Science and Technology lead by Dr. Cheng-Chien Yang synthesized the first composite made of polyaniline and carbon black (PANI-CB)^29^.

It was observed that with increase in the proportion of CB, the conductivity of resultant PANI-CB composite also increased. This increase in conductivity was attributed to the bridging effect of CB, which is entanglement of the conducting back chains of PANI to the surface of CB^30^.

Nonetheless, efforts have been made to alter these composite materials into one dimension through nanostructuring in an effort to enhance their qualities. One-dimensional nanostructures like nanorods, nanowires, and nanotubes have attracted the attention of the scientific community for advanced applications in the fields of electronics and optoelectronic devices because of their novel and intriguing properties^31^.

Lately, current research has been focused on the preparation of nanometric metals dispersed into different polymers^24,25,32,33^. Doping or grafting techniques have been employed to reduce the issues that the other approaches had^25^. Electrochemical approaches can be utilized to regulate the level of coagulation of the disseminated nanoparticles into the polymer matrix^34,35^.

The yield, however, is quite low when using electrochemical methods. Chemical oxidation was used to create PANI nanocomposites containing Pt, Au^25^, and Ag^24^. Because of their improved optical and electrical properties, silver- or gold-containing PANI nanocomposites are among the most significant ones^24^.

Producing polyaniline nanocomposites based on nanocarbon with better electrical and optical properties for various applications has become a challenge in this context. To better understand how the surface shape, particle sizes and composition of these nanocomposites affect their electrical and optical properties, a thorough analysis has been carried out in the current work. The analysis of the relevant electrical properties revealed an innate connection between the electrical performances and the presence of NC and Ag_2_O nanoparticles.

## Methods

### Materials

Aniline (ACS reagent, ≥ 99.5%), silver nitrate (≥ 99.5% (AT)), dodecyl benzene sulfonic acid (DBSA) surfactant are all products of Sigma-Aldrich Company, USA. Carbon black (CB) was provided from Rubber workshop at NRC, Egypt.

### Preparations

#### Preparation of NC

Stable nanocarbon NC transparent solution has been prepared according to Elhalawany et al.^36^ by mixing carbon black CB and DBSA of ratio (1:1) in 80 ml water under continuous vigorous stirring using high shearing effect homogenizer at 10,000 rpm for 10 min till the transparent colloidal solution was obtained.

#### Preparation of polyaniline/NC nanocomposite (PANI/NC)

The specific steps are as follows: In the presence of the previously made NC solution, a 5 × 10^−3^ mol of aniline monomer was polymerized using ammonium persulfate APS as an initiator while being vigorously stirred using high shearing effect homogenizer at 10,000 rpm at room temperature till a green colloidal dispersion characteristic for polyaniline has produced. After being centrifuged, the resulting colloidal dispersion was cleaned, dried, and stored for later use.

#### Preparation of polyaniline/NC/Silver oxide nanocomposite (PANI/NC/Ag_2_O)

The identical processes as for making PANI/NC were followed, but instead of utilising APS, 20 ml of a 1.5 × 10^–2^ M silver nitrate solution was added to the reaction medium while being continuously vigorously stirred using high shearing effect homogenizer at 10,000 rpm at room temperature till the green colloidal dispersion has formed. The produced nanocomposite was ready for use after centrifusion, purification, and drying.

### Characterization

*Infrared spectra* The test has been carried out using FT-IR-6100, Japan. *Transmission electron microscope (TEM)* The test has been done using TEM + DEM Jeol-JEM 1230, Japan. *Scanning electron microscope (SEM)* SEM of the tested Nanocomposites have been done using (QUANTA FEG250) at NRC, Egypt. *Fluorescence Microscopy* The test has been done using fluorescence microscope, with filter cubes A4, I3 and N2.1, Leica Company, Germany, at Microbiology Research Lab., Cairo University. *UV–VIS absorption spectroscopy* The UV–VIS analysis has been done via UviLine 9100 UV–VIS, Germany. *X-ray diffraction (XRD) analysis* XRD analysis has been done using by X-ray diffractometer linked to a computer model PHILIPS-MPD X PERT, wave length: Cu *K*α (*K* = 1.54056 A^o^), USA. *X-ray photoelectron spectroscopy (XPS)* XPS analysis has been done using X-ray photoelectron spectrometer (XPS, Model: KALPHA), under applied pressure 10^–9^ m bar, monochromatic X-ray Al K-alpha radiation from − 10 to 1350 eV and Spot size equals 400 µm. The pass energy was 200 eV and 50 eV for a full spectrum and a narrow spectrum, respectively. *Thermogravimetric analysis (TGA)* TGA has been done using (TGA; PerkinElmer (Germany) at a temperature range of 0–500 °C with a heating rate of 10 °C min^−1^ in a nitrogen atmosphere.

#### Surface analysis

The specific surface area, and pore size distribution of the prepared samples have been determined from nitrogen adsorption–desorption isotherms by a high-speed gas sorption analyzer (NOVA 2000 series, chromatic, UK) at 77 K. Before measurements, the samples were out-gassed at 150 °C in vacuum for 6 h. The pore size distributions were derived from the NLDFT method. The total pore volume was estimated from the amount of gas adsorbed at the maximum relative pressure.

### Cyclic voltammetry measurements

According to Elhalawany et al.^37^, the electrochemical behaviour of the produced nanocomposites was accomplished utilising an Autolab Potentiostat/Galvanostat 302N. Software has been used to monitor the I-V curves. The redox potentials Eox and Ered are calculated using ferrocence material as a reference.

### Electrical measurements

Agilent E4991 B 1 M-1G impedance material analyzer, USA, was used to test the produced nanocomposites' ac conductivity in the frequency range of 0.1 Hz to 10 MHz at room temperature.

## Results and discussion

**Figure 1:**
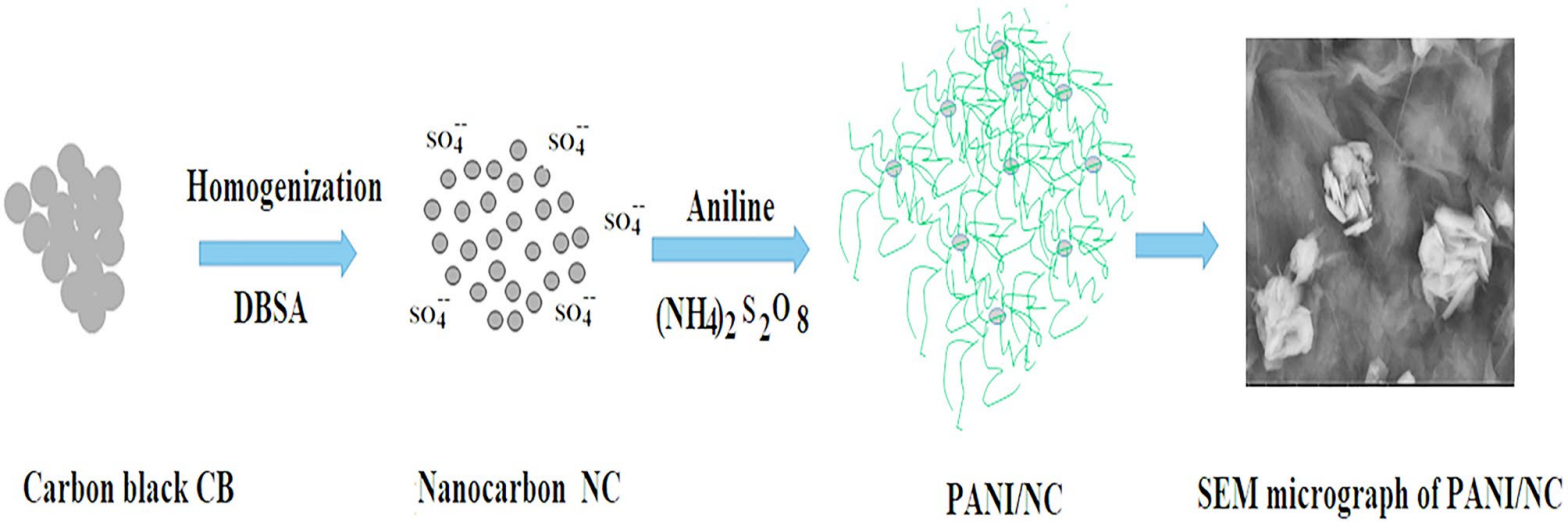
Formation of PANI/NC.

For a variety of applications, PANI/NC and PANI/NC/ Ag_2_O nanocomposites with improved electrical and optical properties have been well prepared in this study effort. There has been put forth a viable mechanism for PANI polymerization in the presence of NC colloidal solution. Here is the proposed mechanism: First, the CB aggregates were dissociated with the help of a powerful high-shearing effect homogenizer at 10,000 rpm to create a stable colloidal solution of nanocarbon NC^36^ that serves as a template for the polymerization of aniline monomer using APS initiator, as shown in Fig. 1. The PANI/NC/Ag_2_O was made in the same manner, with silver ions (Ag +) serving as the initiator.

### Characterization of the prepared nanocomposites

#### FT-IR spectra

**Figure 2:**
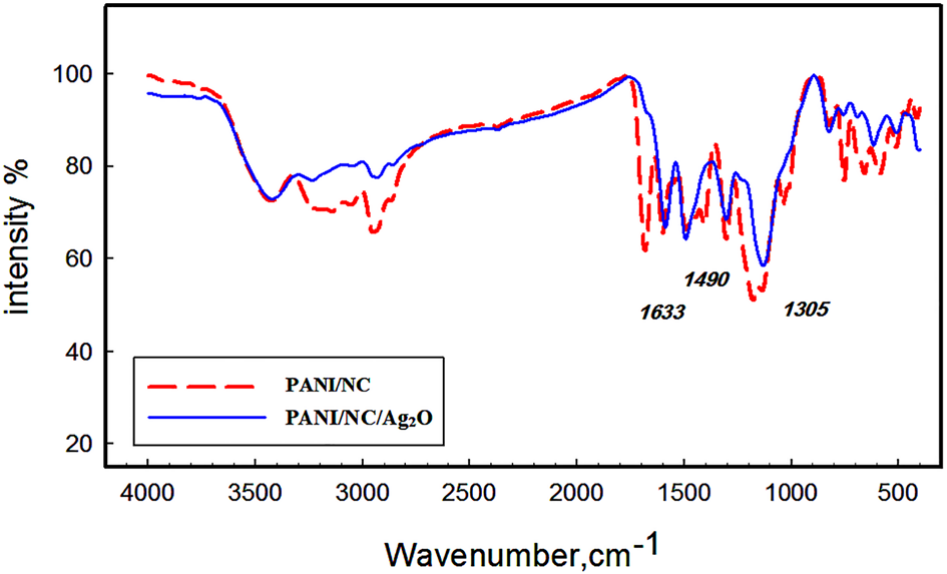
FTIR spectra of the prepared nanocomposites.

The infrared spectra of the PANI/NC and PANI/NC/ Ag_2_O nanocomposites are shown in Fig. 2. As reported in the literature^24,38^ polyaniline has characteristic bands at 1304, 1250, 1374, 1030, 1503, 1633, and 807 cm^-1^. These bands are shown in Fig. 2 but they are slightly shifted owing to composite formation. The main PANI-related absorption peaks can be plainly observed in Fig. 2 at 1503 and 1633 cm^-1^, although they are slightly displaced as a result of composite formation. These are thought to relate to phenazine-like units and o-coupled aniline units^39^. The peak at 1305 cm^-1^, which is a defining feature of the typical PANI base, is attributed to C–N stretching close to a quinonoid ring^24^.

A new peak at 1490 cm^-1^ is also visible in Fig. 2 as a result of the presence of Ag_2_O nanoparticles^24,40^.

#### UV–VIS spectra

**Figure 3:**
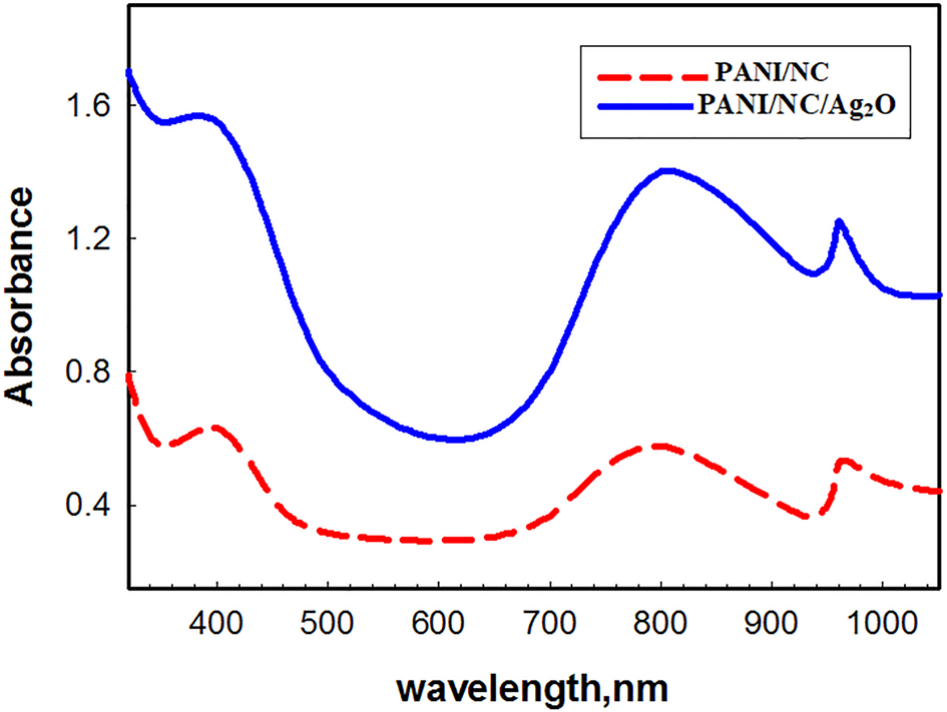
UV–VIS spectra for the prepared nanocomposites.

Figure 3 presents the UV–VIS spectra of the synthesised nanocomposites. The distinctive bands at 430 and 825 nm correspond to transitions that take place in the bezenoid and quinonoid structures of PANI, respectively^41^. A prolonged conjugation, a mixed valence system, or both may be responsible for the appearance of a new peak in the near infrared at 960 nm^42^. The presence of Ag_2_O nanoparticles causes the PANI/NC/ Ag_2_O nanocomposites to exhibit the same peaks with a minor shift. A crucial indicator of the produced nanocomposites' electrical conductivity is their optical band gap (OBG).

According to Tauc's law, the optical band gap OBG may be elucidated from the UV–VIS curve according to the following equation^43,44^:

1 $$ {\upalpha }h\upsilon = (h\upsilon - {\text{E}}_{{\text{g}}} )^{{\text{n}}} $$

where α is the absorption coefficient, *h*υ is the energy of photons, Eg is the optical energy gap OBG and n is the exponent based on the type of electronic transitions.

**Figure 4:**
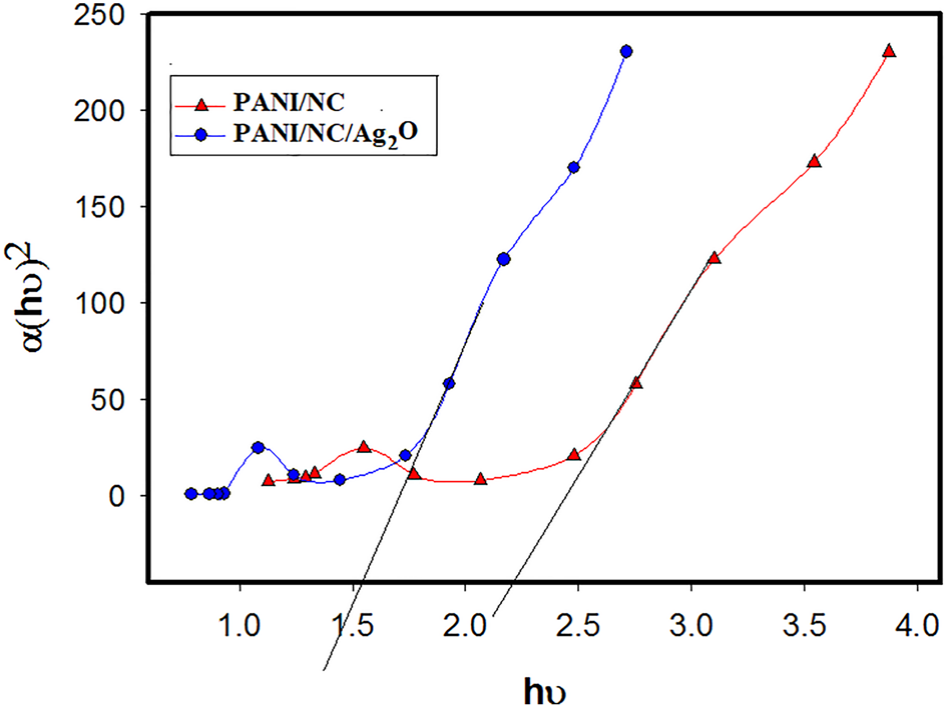
The relation of (α*hυ*)^2^ versus (*hυ*) for the prepared nanocomposites.

The *Eg* values can be obtained from the graph of (α*h*υ)^2^ versus *h*υ as shown in Fig. 4. The OBG has been deduced by continuing the straight line to (a*h*υ)^2^ = 0. The deduced OBG values are 2.1 and 1.5 eV for PANI/NC and PANI/NC/Ag_2_O respectively. The optical band gap OBG has a direct relationship with the conductivity. As shown from the results, the PANI/NC/Ag_2_O nanocomposite has the most extreme results, which indicates improved optical as well as electrical characteristics.

#### Particle size distribution analysis PSD

**Figure 5:**
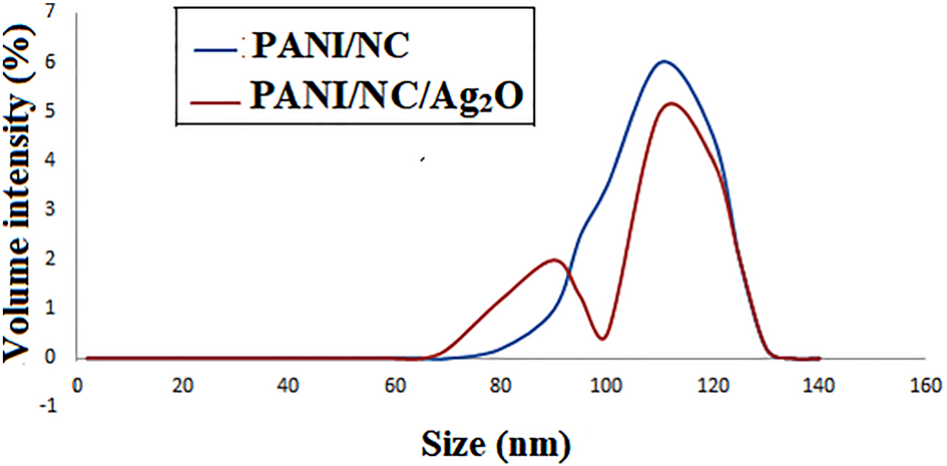
PSD analysis for the prepared nanocomposites.

The percentage of particles of a particular size relative to all particles is known as the particle size distribution. The quantity, volume, etc., of the particle size distribution can be measured. The distribution of particle sizes Fig. 5 illustrates the PSD analysis of the prepared composites. The produced nanocomposites' particle sizes may be shown in the figure to be in the nanoscale range. One broad band in the size range of 80–140 nm is seen in the PSD curve for PANI/NC, whereas two broad bands in the size ranges of 60–100 and 100–140 nm are visible in the PSD curve for PANI/NC/ Ag_2_O.

#### Scanning electron microscope SEM

**Figure 6:**
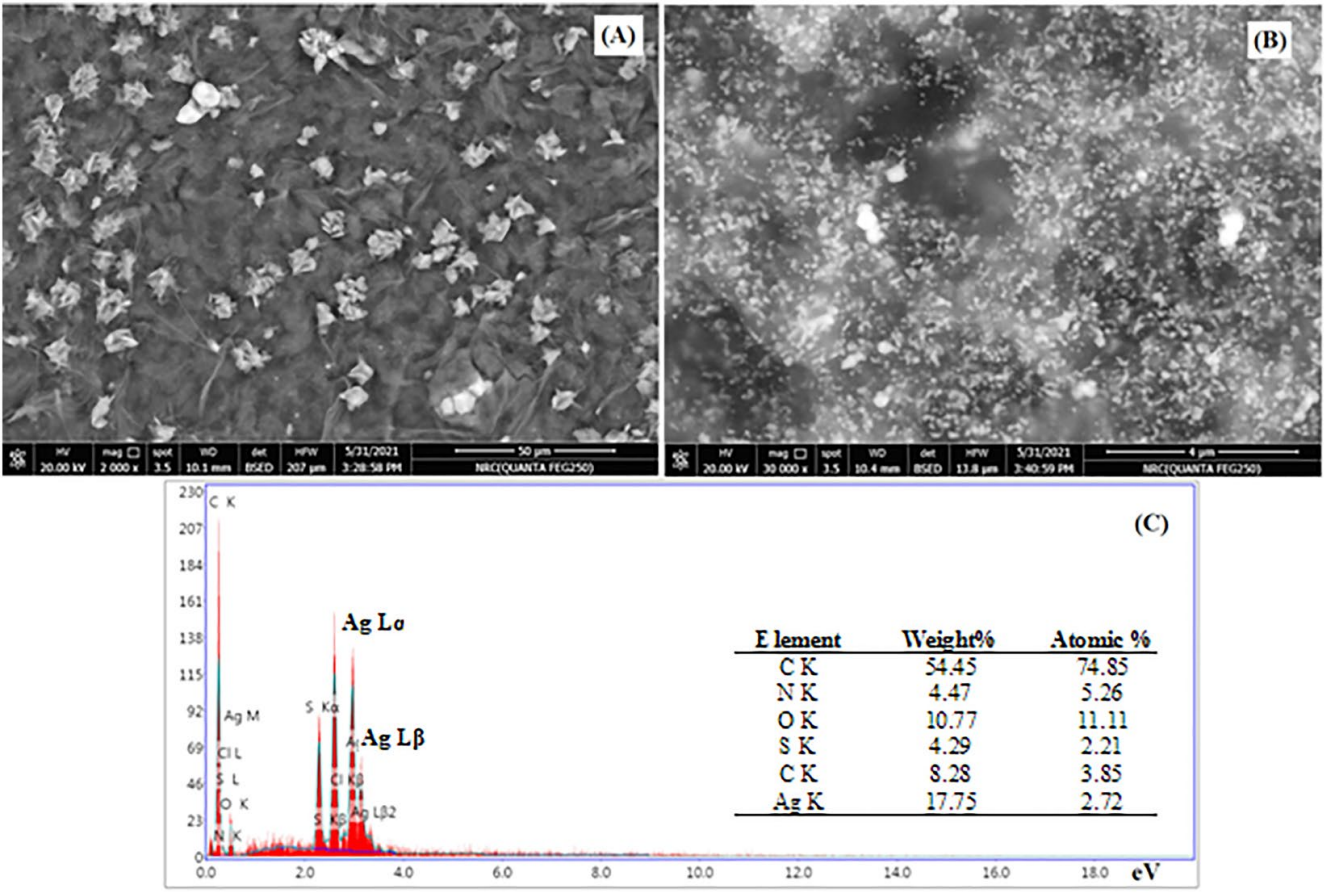
SEM micrograph of (**A**) PANI/NC, (**B**) PANI/NC/Ag_2_O and (**C**) EDX of PANI/NC/Ag_2_O.

Figure 6a–c demonstrate the SEM analysis and energy dispersive x-ray EDX spectra of the prepared nanocomposites. The PANI/NC nanocomposite has adapted the flower-like structures, as seen in Fig. 6a. The deformed structures in Fig. 6b have bright patches that stand in for Ag_2_O nanoparticles. The elemental analysis of PANI/NC/ Ag_2_O using (EDX) is shown in Fig. 6c. Analytical identification of the elements (elemental composition) contained in any given substance is accomplished via energy dispersive X-ray spectroscopy (EDX)^1^.

EDX is a measure to determine the elemental composition of the material. The beam that hits the sample will stimulate an electron in an inner energy level, causing its ejection and hence generating an electron hole. The electron from an outer energy level will be able to jump into this hole accompanied by X-ray emission. The energy of the emitted X-rays will be monitored by the aid of an energy dispersive for elemental composition elucidation. Figure 6c shows the EDX analysis for PANI/NC/Ag_2_O that give rise to a characteristic peak at 2.9 eV, which confirms the presence of silver.

#### Transmission electron microscope TEM

**Figure 7:**
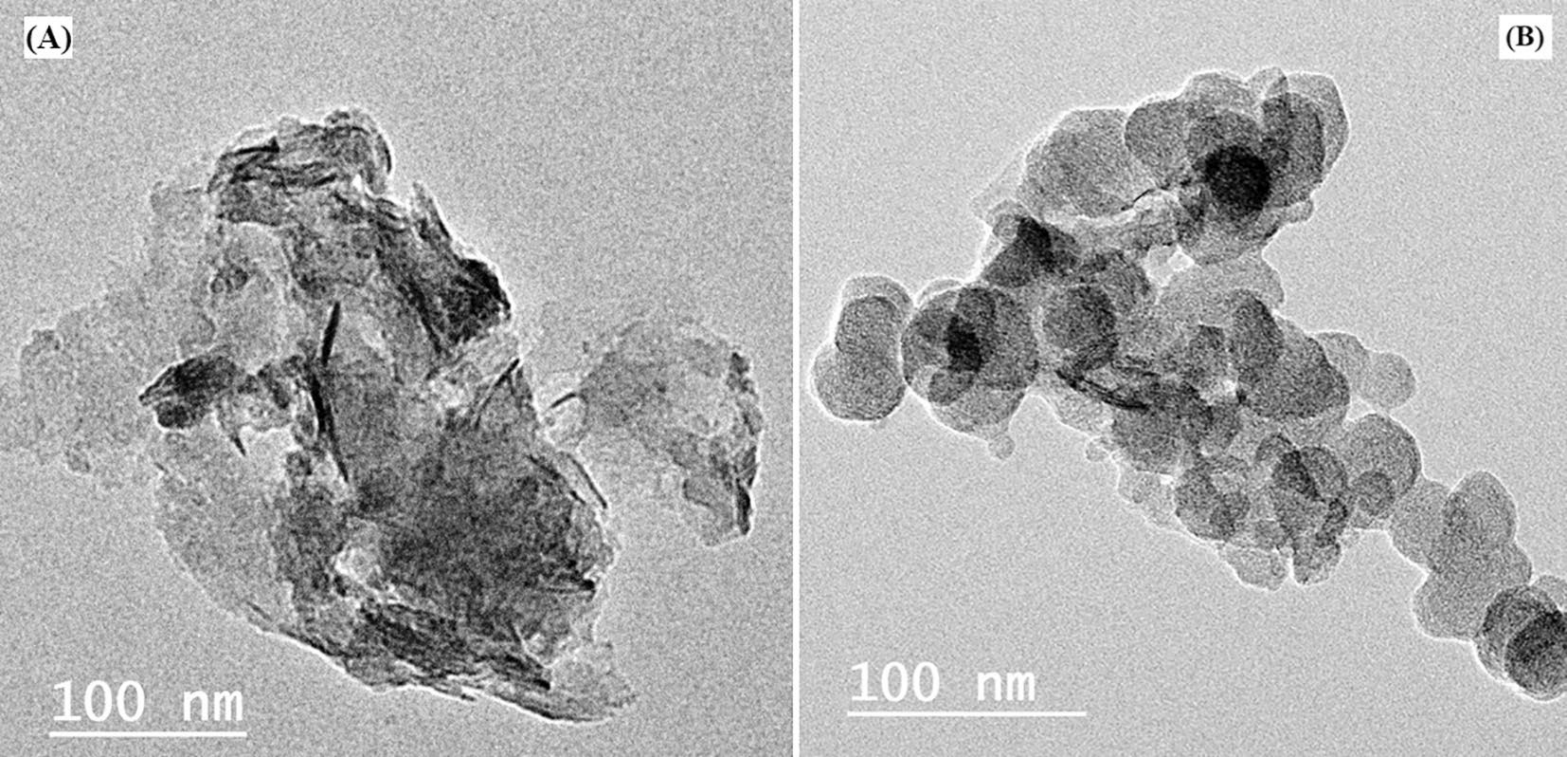
TEM micrograph of (**A**) PANI/NC and (**B**) PANI/NC/Ag_2_O.

The produced composites' TEM graphs are displayed in Figs. 7a,b. As can be seen from Fig. 7a, the NC are randomly distributed across the PANI matrix in the form of black blotches. According to Fig. 7b, the inclusion of Ag_2_O nanoparticles caused the structure to assume the shape of spheres.

#### Fluorescence microscope

**Figure 8:**
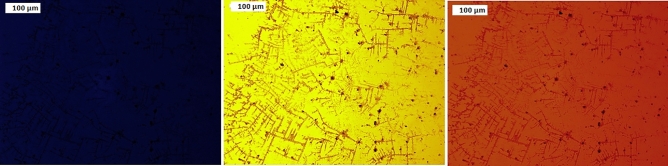
Photoluminescence spectra PANI/NC.

**Figure 9:**
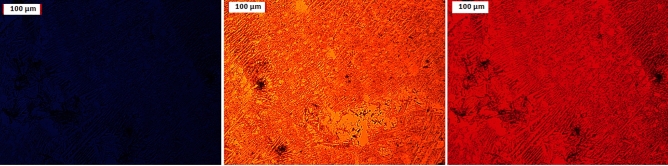
Photoluminescence spectra PANI/NC/Ag_2_O.

Fluorescence is the process through which a substance emits light after absorbing it. The wavelength of the emitted light is often longer than the wavelength of the absorbed light. The current fluorophores, which are in charge of light absorption, absorb light of a specific wavelength and emit light of longer wavelengths when a substance is irradiated by light (i.e., of a dissimilar color from the absorbed one). The optical response of the produced composites to light radiation was examined by the use of a fluorescence microscope examination. The prepared composites are exposed to incident light of different wavelengths from a fluorescence microscope in order to determine the associated changes in their electronic/optical states resulting from charge separation and transport as a result of the incident light on them. Figures 8, 9 show the examination and illustration of the fluorescence characteristics of the created nanocomposites using a fluorescence microscope and three different wave lengths (blue, green, and red). Figures 8, 9 show that the nanocomposites emit nothing when exposed to blue light, yellowish brown when exposed to green light, and orange red when exposed to red light. This suggests that the produced nanocomposites contain fluorophores that are both light-absorbing and capable of longer-wavelength light reemission. These characteristics support their application in LEDs, solar cells, and electronic devices.

#### X-ray diffraction analysis (XRD)

**Figure 10:**
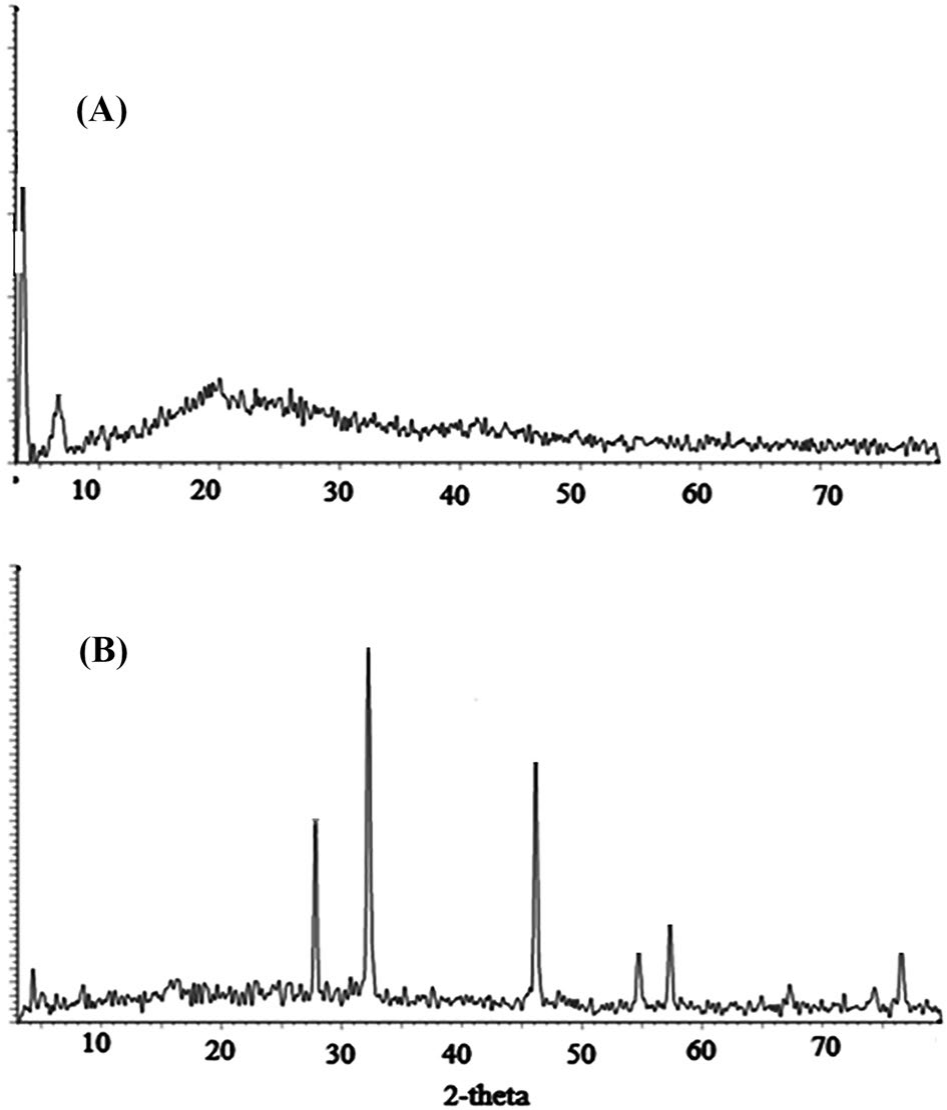
XRD analysis of (**A**) PANI/NC and (**B**) PANI/NC/Ag_2_O.

Figure 10a,b, represents the XRD analysis of PANI/NC and PANI/NC/Ag_2_O. Figure 10a shows broad peak at 2θ = 21 to 25 ^0^ which attributed to the absorption peak for PANI/NC^45^. Figure 10a shows also a small intense peak at 2θ = 6.483 which is due to long PANI chains and more ordered structure^46^. Figure 10b for the PANI/NC/Ag_2_O shows semicrystalline nature. The sharp peaks at 2θ values ~ (32.172, 46.132 and 54.726), may be attributed to the presence of Ag_2_O nanoparticles as reported by Pawar and Wei et al.^47,48^. These results are matchable with the results obtaind from the JCPDS card number 76–1393 of silver oxide.

##### X-ray photoelectron spectroscopy (XPS)

**Figure 11:**
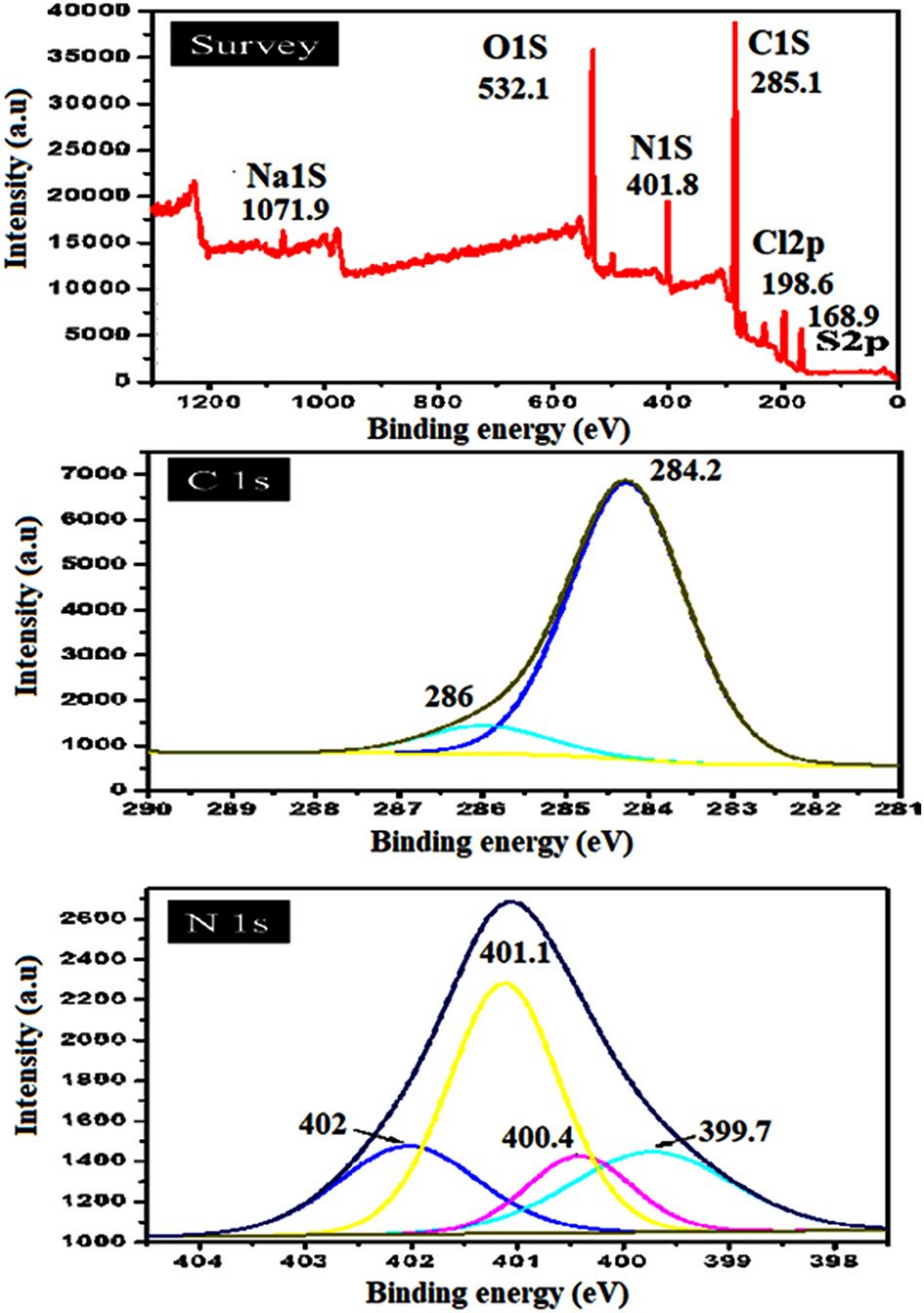
XPS analysis of PANI/NC.

**Figure 12:**
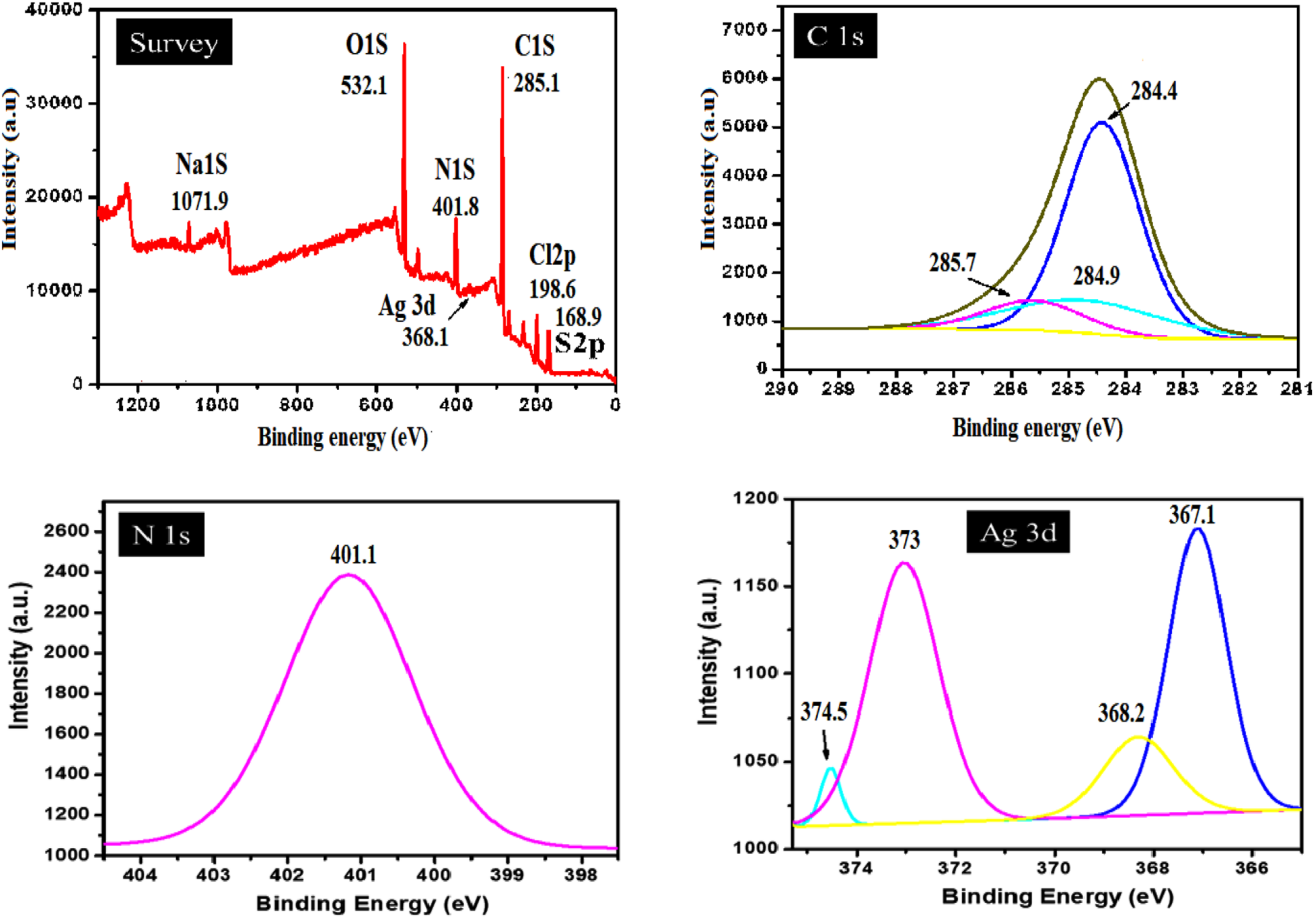
XPS analysis of PANI/NC/Ag_2_O.

Electronic state of carbon in the obtained composites was probed by NEXAFS spectroscopy. The C K-edge spectra of the PANI/NC and PANI/NC/Ag_2_O samples as well as the spectra of individual components are displayed in Figs. 11 and 12. The spectra of initial carbon materials are similar in appearance showing two main peaks located at 285.4 and 291.6 eV corresponding to the π*- and ϭ*-resonances^49^. The C K-edge spectra of PANI/NC in Fig. 11 showed two characteristic peaks at 284.2 eV and at 286 eV corresponding to the carbon atoms bonded to imine nitrogen (= N–) and amine nitrogen (–NH–), respectively^50^. In addition to these peaks the spectrum of the PANI/NC has an intense peak of N at 400.1 eV. NEXAFS investigation of reference compounds has revealed the intense sharp p-resonance between 397.4 and 397.7 eV in the N K-edge spectrum of emeraldine base and two peaks in the ranges 398.8399.1 eV and 402.1402.7 eV in the spectrum of emeraldine salts^51^.

Thus, the peak at 400.1 eV in our spectrum are attributed to imine nitrogen (the splitting of p-resonance arises from conjugation effects^51^, the peak at 399.7 eV can be attributed to the protonated amine groups (–NH^+^–), while the peak around 402 eV is due to transitions within amine nitrogen in –NH– and/or –NH_2_– groups.

According to the literature survey, the location of the XPS peaks of silver depends on the chemical states of silver element and the size of the nanoparticles^52,53^. The binding energy of the.

Ag(0) is higher than that of Ag(I), and the binding energy of Ag(0) in smaller clusters is higher than that in bigger nanoparticles. It was reported that small Ag nanoparticles readily react with oxygen, forming Ag_2_O and AgO upon exposure to air^54^.The binding energies of 368.0–368.3, 367.6–367.8, and 367.3–367.4 eV were reported for pure Ag, Ag_2_O, and AgO, respectively^55,56^.

Figure 12 shows the narrow-range XPS spectra of PANI/NC/Ag_2_O composite. Two peaks for Ag 3*d*5*/*2 were observed at 368.2 and 367.1 eV, representing Ag0 and Ag_2_O particles, respectively, whereas the Ag 3*d*3*/*2 peaks were observed at 374.5 and 373 eV, representing Ag0 and Ag_2_O particles, respectively^57,58^. It is clearly seen from Fig. 12 that the most intensive peaks are located at 367.1 and 373 eV which are corresponding to Ag_2_O nanoparticles. So it is reasonable to draw a conclusion that the formed metallic nanoparticles are mainly composed of Ag_2_O. This result is matchable with XRD analysis.

##### Thermo- gravimetric analysis (TGA)

**Figure 13:**
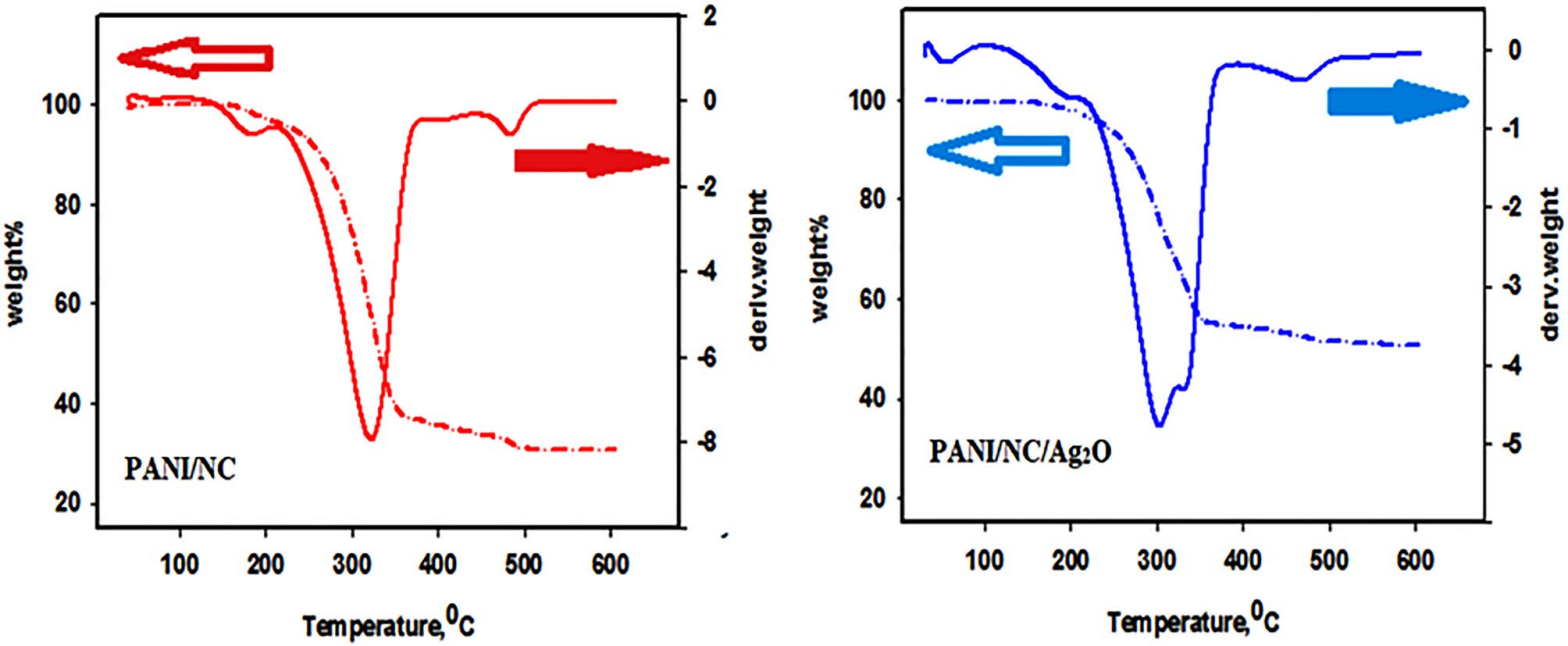
TGA analysis of the prepared nanocomposites.

The thermogravimetric analysis (TGA) of pure PANI/NC and PANI/NC/Ag_2_O nanocomposite is shown in Fig. 13. The nanocomposite has shown stable thermal stability up to a temperature value of 240 °C for PANI/NC and a temperature value of 255 °C for PANI/NC/Ag_2_O. The observed weight losses at temperatures of 240 °C and 255 °C for PANI/NC and PANI/NC/Ag_2_O are 60 and 40 percent, respectively. As shown in Fig. 13, the PANI/NC/Ag_2_O nanocomposite exhibits enhanced thermal stability than that of PANI/NC. The higher thermal stability of the PANI/NC/Ag_2_O nanocomposite may be attributed to the presence of Ag_2_O.

### Surface analysis

**Figure 14:**
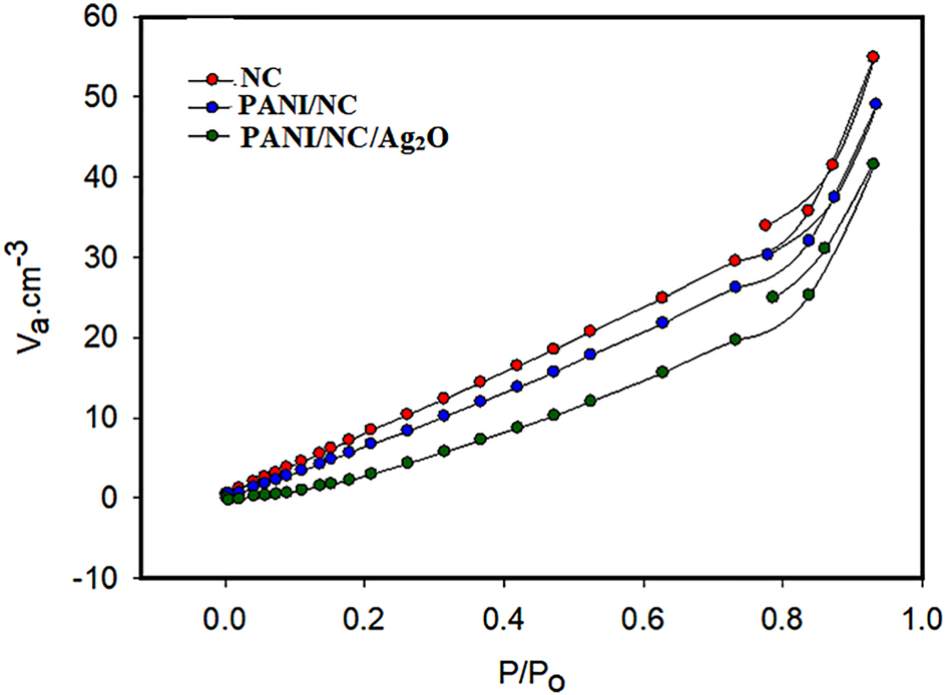
N2 adsorption/desorption isotherms for the prepared materials.

**Figure 15:**
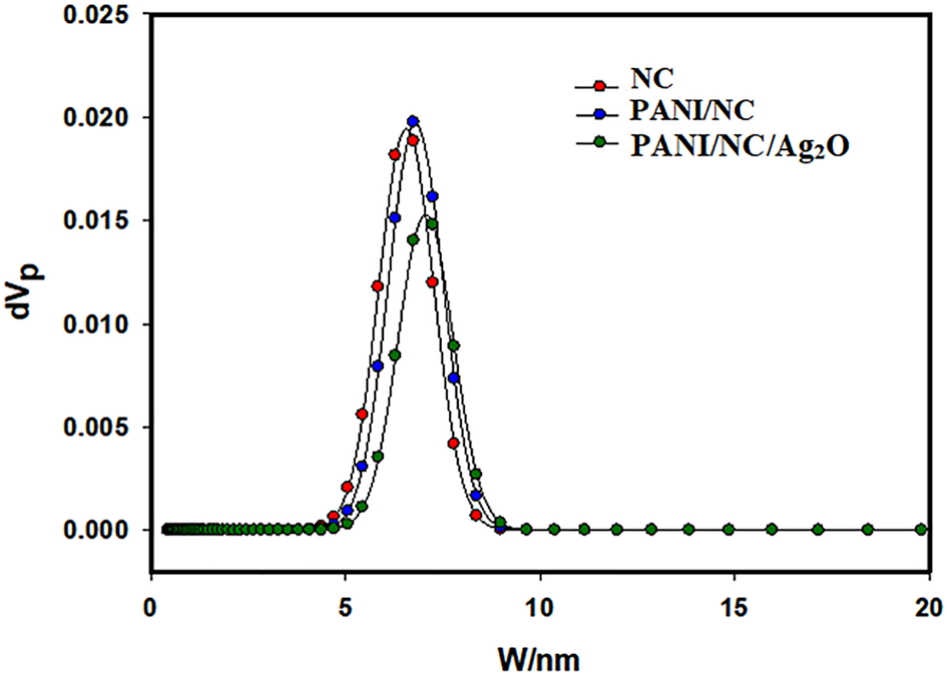
NLDFT/GCMC pore size distribution analysis.

The superiority of the materials is often specified by their surface area (SA), pore volume, mean pore diameter, and pore size distribution. The nitrogen isotherm data of the tested samples are displayed in Fig. 14. The samples are shown to be mesoporous in structure, and the adsorption curve seems to be an IUPAC type II curve. The pore size distributions (PSDs) of the samples under study are displayed in Fig. 15.

According to IUPAC, pores with an internal width of less than 2 nm, in the range of 2–50 nm, and greater than 50 nm are considered micropores, mesopores, and macropores, respectively^36^. From the PSDs data displayed in Fig. 15, one can conclude that the tested samples are mesoporous.

Table 1 outlines the estimated porous parameters of the samples. It can be seeing that NC sample has the highest surface area SA and the lowest pore size PS. However, the SA and the PS of the PANI/NC and PANI/NC/Ag_2_O samples are decreased slightly. This finding indicated that the presence of PANI or PANI/Ag_2_O doesn’t affect the mesoporous structure of NC.

**Table1:** The estimated porous parameters of the prepared materials.

Samples	SSA (m^2^/g)	Total pore volume (cm^3^/g)	Mean pore diameter (nm)	Maximum pore size (nm) from NLDFT
NC	56.8	0.0848	5.97	6.4
PANI/NC	51.3	0.0758	5.90	6.75
PANI/NC/Ag_2_O	14.8	0.0643	17.4	7.25

### Cyclic Voltammetric measurements

By measuring the oxidation and reduction potentials (E_ox_ & E_red_) of the chemical material, cyclic voltammetry (Cv) plays a significant role in monitoring the oxidation and reduction reactions. Thus, using the recorded data, the energy band gap E_g_ can be determined (E_ox_ & E_red_). The result of subtracting the energies of the highest occupied molecular orbital (HOMO) and the lowest unoccupied molecular orbital (LUMO) yields the energy band gap E_g_.

The HOMO is regarded in solid state physics as the energy needed to eliminate an electron by oxidation. The LUMO is the opposite, since it provides the energy required for the reduction process, which inserts an electron. Cyclic voltammetric measurements can be used to analyze these oxidation–reduction processes.

**Figure 16:**
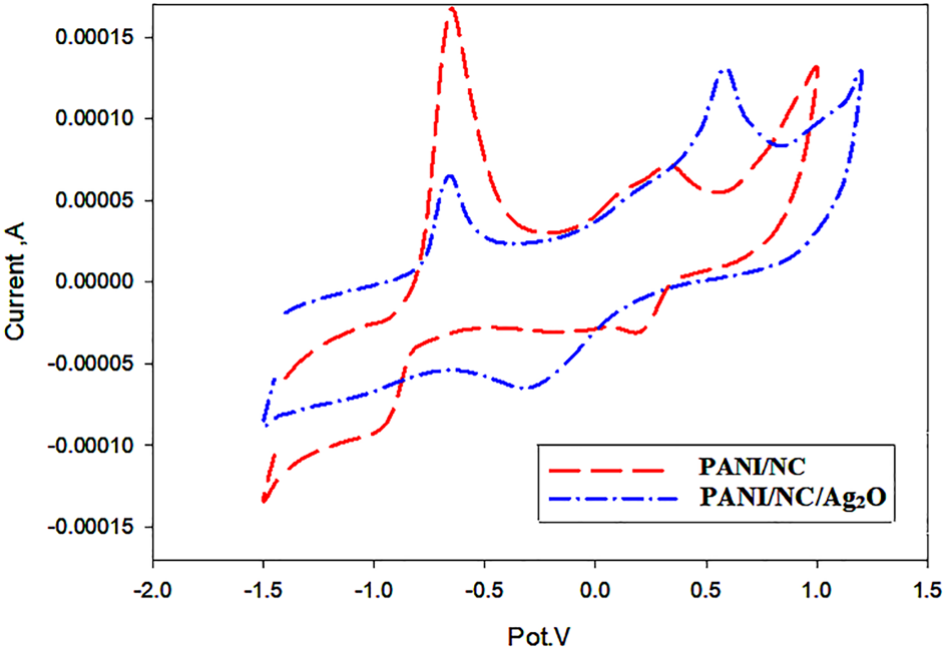
Cyclic voltammogram of the prepared nanocomposites.

The prepared nanocomposites' cyclic voltammogram CV of the current–voltage I–V curve is displayed in Fig. 16. Due to the electrochemical oxidation and reduction events that took place, Fig. 16 depicts typical peaks in the anodic and cathodic branches of the CV curve for PANI/NC and PANI/NC/Ag_2_O, respectively.

The positive shift in the anodic potential observed for PANI/NC/Ag_2_O suggests chemical interactions between the chain parts of PANI/NC and the present Ag_2_O nanoparticles.

E_HOMO_ and E _LUMO_ can be elucidated from the following equations^24^:

2 $$ {\text{E}}_{{{\text{HOMO}}}} = [\left( {{\text{E}}_{{{\text{ox}}}} {\text{onset }}\left( {{\text{vs}}.{\text{ Ag}}/{\text{AgCl}}{-}{\text{E}}_{{{\text{onset}}}} \left( {{\text{Fc}}/{\text{Fc}} + {\text{vs}}.{\text{ Ag}}/{\text{AgCl }}} \right)} \right) + {4}.{8}} \right]{\text{ eV}}. $$

3 $$ {\text{E}}_{{{\text{LUMO}}}} = [\left( {{\text{E}}_{{{\text{red}}}} {\text{onset}}\left( {{\text{vs}}.{\text{ Ag}}/{\text{AgCl}}{-}{\text{E}}_{{{\text{onset}}}} \left( {{\text{Fc}}/{\text{Fc}} + {\text{vs}}.{\text{ Ag}}/{\text{AgCl}}} \right)} \right) + {4}.{8}} \right]{\text{ eV}}. $$

E_HOMO_ and E_LUMO_ for PANI/NC are 4.83 eV and 3.62 eV and that for PANI/NC/Ag_2_O are 4.7 eV and 3.72 eV respectively. The energy band gap E_g_ can be assumed to be 1.21 and 0.98 eV for PANI/NC and PANI/NC/ Ag_2_O, respectively, because it is equal to the difference between the HOMO and LUMO energies. These prominent electrical and optical characteristics are indicated by the low E_g_ values.

All polymers' electrochemical energy band gaps E_g_ are invariably less than those estimated from UV–VIS spectra^59^. Although it has been studied in the literature, the discrepancy between the energy gab E_g_ and the optical energy gap E_opt_ still requires a full study.

### Electrical conductivity measurements

Conjugated polymers' conductivity is one of their most important features for a variety of applications. Such polymers' conductivity is attributed to the generation of polarons and bipolarons, the dominant charge carriers. The radical cation with the accompanying lattice distortion that creates a positively charged hole site is known as a polaron. This hole site is conductive due to its mobility within the polymer conjugation system. These polymers often use charge carrier hopping as their conduction mechanism^24,32^.

Conducting polymers typically have an energy gap Eg separating their occupied valence band from their vacant conduction band. These polymers can be doped to improve the oxidation–reduction processes, which leads to the formation of new energy levels in the energy gap and hence enhancement in conductivity^60^.

Carbon black’s filled polymers are considered conductors; at room temperature, their conductivity ranges from 0.1 to 102 S/cm^1^. Above a certain concentration, carbon black-filled polymers become conductors. As a result, carbon blacks are frequently utilised as fillers in polymers for uses including conductive packaging for electronic components and conductor shield composites for cables^2^.

The ac conductivity of synthesized pure PANI/NC and PANI/NC/Ag_2_O at room temperature has been examined at frequency ranges from 0.1 Hz to 10 MHz. Capacitance Cp and conductance G values have been noted using the LCR Bridge.

**Figure 17:**
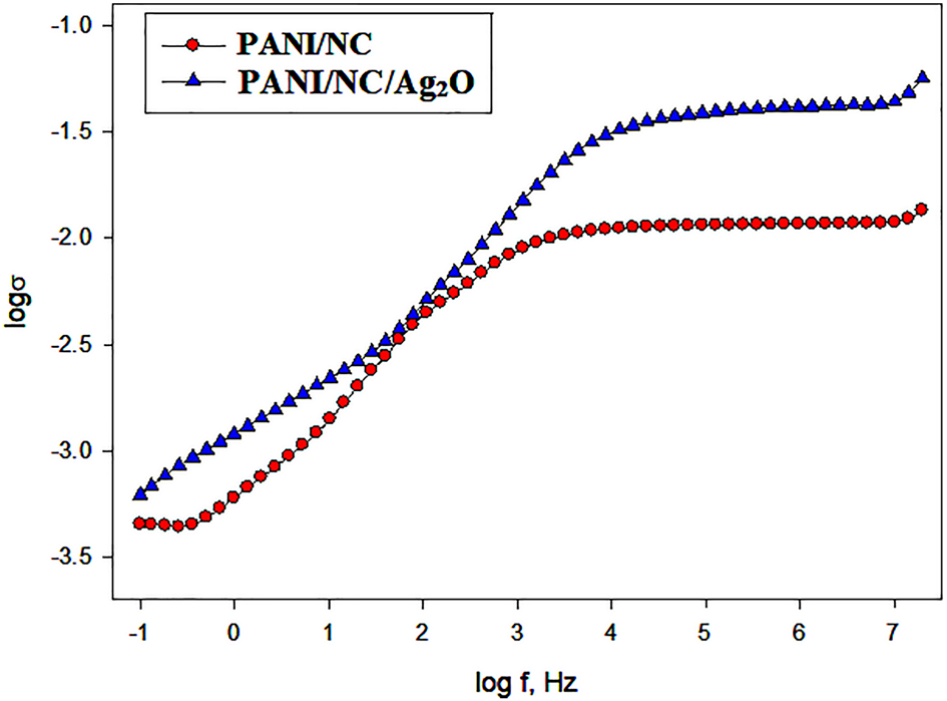
Variation of AC conductivity with angular frequency for the prepared nanocomposites.

At room temperature, Fig. 17 depicts the relationship between ac conductivity fluctuation and angular frequency variation for the synthesized nanocomposites. Figure 17 showed that the ac conductivity rose with frequency and was significantly higher in the silver-doped nanocomposite (PANI/NC/Ag_2_O) than in PANI/NC. Ag_2_O nanoparticles are thought to be responsible for this increase in ac conductivity since they make charge transfer much easier.

And as predicted by XRD analysis, Ag_2_O nanoparticles have increased the crystallinity of the resulting composite, and as a result, their chemical structure has become more ordered and their electrical conductivity has increased^61^.

According to Fig. 17, the maximum ac conductivities for the PANI/NC and PANI/NC/ Ag_2_O nanocomposites are 1.06 × 10^–2^ and 2.5 × 10^–2^ S.cm^-1^ respectively.

The development of new energy levels in the band gap area, which makes it easier to transmit charges, is the likely cause of this increase in conductivity^62^.

In general, the following equation describes how the ac conductivity of polymers evolves with frequency:

4 $$ {\upsigma }_{{{\text{ac}}}} \left( \omega \right) = {\text{A}}\omega^{s} $$

where A is a constant that is typically temperature-dependent, ω is the angular frequency (ω = 2*π*f) and S is the frequency exponent, which typically falls between 0 and 1.

Figure 17's straight line slopes can be used to compute the frequency exponent (s). For PANI/NC and PANI/NC/ Ag_2_O nanocomposites, respectively, the s values are 0.35 and 0.46.

The hopping mechanism and the observed behaviour of (S) are compatible. According to the hopping model, electrical conduction happens through a process called bipolaron hopping, in which two polarons instantly hop or jump over a potential barrier between two charged states. The height of the barrier is related to the separation of the formed polarons via a columbic interaction. This is consistent with our findings, which show that the inclusion of NC and Ag_2_O nanoparticles improves the transitions of the polarons that are generated and, as a result, increases conductivity.

### Dielectric properties

**Figure 18:**
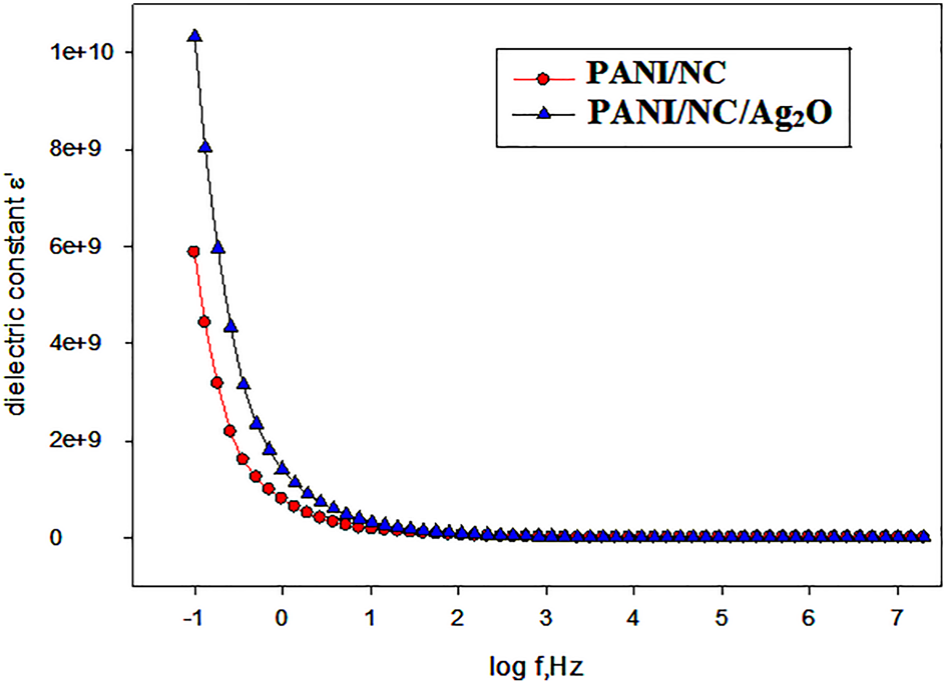
Variation of dielectric constant with angular frequency for the prepared nanocomposites.

The measured capacitance value has been used to compute the dielectric constant at room temperature. The following equation was used to get the dielectric constant (ε′):

5 $$ \varepsilon^{\prime} = C_{p} /A(\varepsilon^{o} ) $$

where A is the cross sectional area, (ε^ο^) is the free space permittivity, and Cp is the capacitance. Figure 18 depicts the relationship between the dielectric constant (ε′) and angular frequency Ln(ω). Figure 18 demonstrates that (ε′) drops as frequency increases. This may be attributed to the inability of the formed dipoles to adapt themselves to be in the same direction of the applied electric field^32^. As a result, it is anticipated that the tendency for polarization to decrease with increasing frequency will also occur.

The results revealed that the dielectric constant (ε′) of the silver dopped nanocomposite is much higher than that of PANI/NC at all frequencies. This increase is attributed to molecules’ polarizations when exposed to an applied electric field, where the dielectric permittivity of the material depends on the extent of polarization of the molecules^63^.

It can be concluded that the existence of NC and Ag_2_O nanoparticles in the formed composites enhances the dielectric permittivity due to enhanced polarization.

The findings demonstrated that at all frequencies, the dielectric constant (ε′) of the silver-doped nanocomposite is much higher than that of PANI/NC. This rise is attributable to the polarisation of the molecules in an electric field, where the degree of polarisation of the molecules determines the material's dielectric permittivity, i.e., the greater the degree of polarisation, the higher the permittivity^63^.

One can conclude that the interfacial polarisation that results in an increase in dielectric permittivity is due to the inclusion of NC and Ag_2_O nanoparticles in the produced composites to enhance the dielectric permittivity.

## Conclusion

The successful preparation of PANI/NC and PANI/NC/ Ag_2_O nanocomposites has been accomplished employing a high shearing effect homogenizer and a chemical oxidative approach. According to the PSD and TEM analysis the sizes of the prepared nanocomposites are in the range of 60–140 nm. The EDX analysis revealed a typical silver nanoparticle peak, confirming the doping procedure. XPS and XRD analysis confirmed the presence of Ag_2_O nanoparticles. According to the observations made using a fluorescence microscope, the produced nanocomposites displayed luminescence when exposed to various wavelengths of light. This suggests that the prepared nanocomposites contain fluorophores with the ability to both absorb and emit light. The maximum ac conductivities for the PANI/NC and PANI/NC/ Ag_2_O nanocomposites, respectively, were 1.06 × 10^–2^ and 2.5 × 10^–2^ S.cm^-1^. A concept for their future usage in optoelectric devices like solar cells is generated by their improved optical and electrical capabilities. Finally, it can be said that the created nanocomposites are characterized by easy processing and low cost, and this will open up new possibilities for the technology of nanocomposites and their many applications, particularly in optoelectric devices like solar cells.

## Data Availability

The datasets used and/or analysed during the current study available from the corresponding author on reasonable request.
